# Myelination is delayed during postnatal brain development in the *mdx* mouse model of Duchenne muscular dystrophy

**DOI:** 10.1186/s12868-017-0381-0

**Published:** 2017-08-14

**Authors:** Azeez Aranmolate, Nathaniel Tse, Holly Colognato

**Affiliations:** Department of Pharmacological Sciences, Stony Brook University, Stony Brook, NY 11794-8651 USA

**Keywords:** Duchenne muscular dystrophy, Dystrophin, Oligodendrocyte, Myelin

## Abstract

**Background:**

In Duchenne muscular dystrophy (DMD), the loss of the dystrophin component of the dystrophin-glycoprotein complex (DGC) compromises plasma membrane integrity in skeletal muscle, resulting in extensive muscle degeneration. In addition, many DMD patients exhibit brain deficits in which the cellular etiology remains poorly understood. We recently found that dystroglycan, a receptor component of the DGC that binds intracellularly to dystrophin, regulates the development of oligodendrocytes, the myelinating glial cells of the brain.

**Results:**

We investigated whether dystrophin contributes to oligodendroglial function and brain myelination. We found that oligodendrocytes express up to three dystrophin isoforms, in conjunction with classic DGC components, which are developmentally regulated during differentiation and in response to extracellular matrix engagement. We found that *mdx* mice, a model of DMD lacking expression of the largest dystrophin isoform, have delayed myelination and inappropriate oligodendrocyte progenitor proliferation in the cerebral cortex. When we prevented the expression of all oligodendroglial dystrophin isoforms in cultured oligodendrocytes using RNA interference, we found that later stages of oligodendrocyte maturation were significantly delayed, similar to *mdx* phenotypes in the developing brain.

**Conclusions:**

We find that dystrophin is expressed in oligodendrocytes and influences developmental myelination, which provides new insight into potential cellular contributors to brain dysfunction associated with DMD.

**Electronic supplementary material:**

The online version of this article (doi:10.1186/s12868-017-0381-0) contains supplementary material, which is available to authorized users.

## Background

Duchenne muscular dystrophy (DMD), resulting from mutations in the dystrophin gene on the X chromosome, is characterized by muscle degeneration and premature death [1]. A large subset of DMD patients also have neurodevelopmental problems, of which the cellular basis remains poorly understood, a problem that is compounded by the limited understanding of dystrophin function in the central nervous system (CNS). Dystrophin is part of the dystrophin-associated glycoprotein complex, or DGC, which connects the extracellular matrix to the cytoskeleton and provides a regulatory hub for signaling pathways [2, 3]. The canonical DGC consists of extracellular laminin, dystrophin, dystroglycan, sarcoglycans, dystrobrevins, syntrophins and, sometimes, either utrophin or dystrophin-related protein 2 [4]. Although best characterized in muscle, dystrophin and putative DGC components have been found in many other tissues [4–9], including the brain. However it remains unknown whether oligodendrocytes, the myelinating cells of the CNS, also contain dystrophin and an associated DGC. As correctly executed and timed myelination is critical for the speed and coordinated timing exhibited in complex brain processing [10, 11], it is possible that the loss of oligodendroglial dystrophin could contribute to brain dysfunction seen in DMD.

The dystrophin gene contains 79 exons and seven tightly regulated internal promoters, which produce multiple protein isoforms with diverse expression in tissues. While the five main dystrophin isoforms differ in the N-terminal region and length of the central rod domains [9, 12], they all share the same C-terminal region that binds dystroglycan, a transmembrane receptor that links the DGC to the extracellular matrix. Several studies have linked the loss of dystrophin expression to cognitive impairments in DMD, with a higher propensity for these impairments being observed in mutations that result in the loss of multiple isoforms [9, 12–15]. DMD patients have smaller total brain volume, smaller gray matter volume, lower fractional anisotropy and higher white matter diffusivity [12]. Some DMD patients have intellectual disability and/or other cognitive impairments such as learning disorders (e.g., dyslexia), attention deficits (e.g., ADHD), autism, seizures, and neuropsychiatric disorders [9, 12, 16]. In *mdx* mice, the best characterized model of DMD, expression of the largest isoform of dystrophin (Dp427) is lost, similar to what occurs in the majority of DMD patients. Currently, the underlying cellular changes that contribute to DMD cognitive deficits remain unclear, although both hippocampal synaptic dysfunction and blood brain barrier disturbances have been identified in *mdx* mice [17, 18]. *mdx* mice also have enlarged lateral ventricles, elevated diffusion in the prefrontal cortex and reduced fractional anisotropy in the hippocampus [19], the latter two changes being suggestive of potential myelin abnormalities.

Dystrophin’s binding partner, dystroglycan, and dystroglycan’s extracellular ligand, laminin, have both been implicated in regulating CNS myelination. For example, a global loss of the laminin alpha2 subunit leads to myelination deficits or delays [20–22]. And, selective dystroglycan loss in oligodendocytes reduces their ability to differentiate [23–25], and delays myelination in the brain [26]. Given the important role of myelination in many of the brain processes affected in DMD, and the connection between dystroglycan and myelination, the goal of the current study was to determine if dystrophin regulates CNS myelination. We characterized for the first time an oligodendroglial DGC, which is both developmentally and ECM-regulated during oligodendrocyte maturation. We further identified three distinct dystrophin isoforms in oligodendrocytes, whose levels were developmentally regulated. We subsequently found that *mdx* mice had delayed myelination, primarily in the cerebral cortex, which was accompanied by higher than normal densities of proliferative oligodendrocyte progenitors. To address whether myelination defects were likely caused by dystrophin loss in oligodendrocytes themselves, we prevented the expression of all dystrophin isoforms in primary oligodendrocytes and found a similar delay in oligodendrocyte maturation. The current study demonstrates a new role for dystrophin in achieving successful developmental myelination, suggesting that the loss of oligodendroglial dystrophin(s) may be a contributing factor in DMD-related neurological deficits.

## Methods

### Rat primary oligodendrocyte progenitor cell cultures

Timed-pregnant female Sprague–Dawley rats were obtained from Harlan Laboratories-Envigo (IL, USA). The neonatal cerebral cortices of P0–P2 pups were dissected, cortical meninges removed, and cortices digested using papain (Worthington, Lakewood, NJ, USA) mixed in a high-glucose Dulbecco’s modified Eagle’s medium (Corning Cellgro, VA, USA), supplemented with penicillin, streptomycin and 10% fetal bovine serum. Mixed glial cultures were prepared by seeding digested cortices onto poly-d-lysine (PDL)-coated flasks incubated at 37 °C, 7.5% CO_2_ for 10–14 days, with media changes approximately every 3 days. Oligodendrocyte progenitor cells (OPCs), were purified using a modification of the classical mechanical dissociation and differential adhesion method [23, 27]. Isolated OPCs were plated on two substrates: 5 µg/mL poly-d-lysine diluted in water (PDL; Sigma, USA), which is permissive for OPC attachment, or 10 µg/mL laminin (Purified human merosin; #CC085, Millipore Corporation, MA, USA), a ligand for dystroglycan, diluted in ice-cold phosphate-buffered saline (PBS). OPCs were harvested after 1, 3 and 5 days in either proliferation (Sato’s medium plus 10 ng/mL platelet-derived growth factor and 10 ng/mL basic fibroblast growth factor; Peprotech, NJ, USA) or differentiation medium (Sato’s medium plus 0.5% FBS). These time points were selected based on expected maturation in differentiation-inducing medium: day 1 generates OPCs that have begun to exit the cell cycle and some newly-formed oligodendrocytes, day 3 generates immature oligodendrocytes, and day 5 generates mature oligodendrocytes [28].

### Primers

Dystroglycan (Accession#: XM_343483.4, Fwd: TGGCACGGCTGTTGTTGGGC, Rev: CCTTCCCTGCTGCAGACACCTTG). Dystrophin 71c (Pan) (Accession#: NM_001005246.1, Fwd: GGGGACAACATGGAAACAAATCTGC, Rev: GAGGAGACGGCAGTGGGGACA). Utrophin (Accession#: NM_013070.1, Fwd: CCCGAACACGCCCAGAGGAAC, Rev: GGTGCCCGTACTTGGCCATC). Syntrophin B1 (Accession#: NM_001130542.1, Fwd: GCTGAAATCACCACCGCCTGC, Rev: GGCTTGGGGCAGGAGTGAAGG). Syntrophin B2 (Accession#: NM_001168674.1, Fwd: CTGGCTGGCAGAGCAGGCAAAA, Rev: CCAAGGGAAGGCGACCGACA). Dystrobrevin Alpha (Accession#: XM_017601078.1, Fwd: GTGAGCCTTTGCACCCCATGTT, Rev: TGCGATCAGCTGCCTTTGCTGT). Dystrobrevin Beta (Accession#: NM_001012191.1, Fwd: ACTGCATGGACACACGCCCG, Rev: GGTGCCAGCTTGCTCCTCCG). Sarcoglycan Alpha (Accession#: NM_001107039.1, Fwd: CGG CGT GAA GGA CGG CTG AAG, Rev: GTG GAT AGA GGC CGG GGC AC). Sarcoglycan Beta (Accession#: NM_001191068.1, Fwd: CCGAGCAGCAAGGTTCCAACG, Rev: CGTTCCACTGCCTTCTCCCGC). Sarcoglycan Delta (Accession#: NM_001109029.1 & NM_001134826.1, Fwd: GAACAGTACTCACACCACAGGAG, Rev: GCATTACCTGGTCGGGACTTGAT). Sarcoglycan Epsilon (Accession#: NM_001002023.2, Fwd: AGCCCCCTTCGGATTCTTTG, Rev: TCTTTTCCACTCCTTCCCGTC). Sarcoglycan Gamma (Accession#: NM_001006993.1, Fwd: CGCTGTTCACCGCCGAGGAG, Rev: GCATCGAGCACCAGCACTCCA). Sarcoglycan Zeta (Accession#: NM_001108875.2, Fwd: GGACAGCTGACAGTCGGAGCTGAG, Rev: CAGCACCCTGCCGTCTTCACT). Syntrophin A1 (Accession#: NM_001100901.1, Fwd: GGCATCAGCATCAAGGGAGG, Rev: TGGGTGGCAGAGGACAAATC). Syntrophin Gamma1 (Accession#: NM_001191981.1, Fwd: GAAGACTGTGCATGTGCTCC, Rev: AAAGCGGGCATGTAGTAGGG). Syntrophin Gamma2 (Accession#: NM_001106720.1, Fwd: GGTGCACATGGGATGGGTAA, Rev: CAGTGTGCTTACCGGAGGAG). Dystrophin related protein 2 (Accession#: NM_023971.1, Fwd: GCCCCCTGGAACCATCAGCC, Rev: GCGAGCGCGGAGGTTGTGAG). Glyceraldehyde 3-phosphate dehydrogenase (GAPDH) [29]. Mdx-Common Forward (DL1577; GCGCGAAACTCATCAAATATGCGTGTTAGTGT) [30]. Mdx-Wildtype Reverse (DL1509; GATACGCTGCTTTAATGCCTTTAGTCACTCAGATAGTTGAAGCCATTTTG) [30]. Mdx-Mutant Reverse (DL1573; CGGCCTGTCACTCAGATAGTTGAAGCCATTTTA) [30].

### Antibodies

Mouse IgM anti-α-Dystroglycan (clone IIH6C4, IHC 1:100, Millipore Corporation, USA). Mouse anti-β-Dystroglycan (MANDAG2, WB 1:50, IHC 1:10, Developmental Studies Hybridoma Bank, USA). Mouse anti-Dystrophin (C-term, WB 1:50, IHC 1:250, Millipore, USA). Mouse anti-Dystrophin (C-terminal, WB 1:50, Leica Novocastra, UK). Mouse anti-Dystrophin (Rod domain, WB 1:250, Millipore, USA). Rabbit anti-Utrophin (H-300, IHC 1:100, Santa Cruz Biotechnology, USA). Rat anti-Myelin Basic Protein (MBP 82-87, WB 1:5000, IHC 1:100, Serotec Inc, USA). Mouse anti-β-Actin (WB 1:10,000, Sigma, USA). Mouse anti-p115 (WB 1:5000, BD Biosciences, USA). Rabbit anti-Platelet-derived growth factor receptor, alpha (PDGFRα C-20, IHC 1:100, Santa Cruz biotechnology, USA). Mouse anti-2′,3′-cyclic nucleotide 3′-phosphodiesterase (CNPase, Clone 11-5B, WB 1:1000, IHC 1:100, Sigma, USA). Mouse anti-APC/CC1 (Ab7, IHC 1:100, Calbiochem, USA). Rabbit anti-Olig2 (18953, IHC 1:100, Immuno-Biological Labs, Japan). Rat anti-Mouse/Rat Ki67 (Sol A1, IHC 1:250, eBioscience, USA). Rabbit anti-LaminB1 (ab16048, WB 1:5000, Abcam, UK). Rabbit anti-ENPP6 (ab127897, Abcam). Rabbit anti-cleaved caspase-3 (Asp175, #9661, Cell Signaling Technologies). LI-COR infrared-coupled secondary antibodies (IR-800; WB 1:10,000, Rockland Inc, USA). FITC-conjugated donkey anti-rabbit IgG (IHC 1:200, Jackson ImmunoResearch, USA). Cy3-conjugated donkey anti-mouse IgG (IHC 1:500, Jackson ImmunoResearch). Biotin-SP-conjugated F(ab′)^2^ donkey anti-mouse IgM (IHC 1:200, Jackson ImmunoResearch, USA) and Cy3-conjugated streptavidin (IHC 1:500, Jackson ImmunoResearch, USA) were used to detect α-Dystroglycan.

### Real-time qRT-PCR

An RNeasy mini kit (Qiagen) was used to purify RNA from oligodendrocytes. Equal amounts of RNA from each sample was converted to cDNA using a ProtoScript II First Strand cDNA Synthesis Kit (New England Biolabs). The gene products were then amplified using Fast SYBR Green master mix (Applied Biosystems). Oligodendroglia were analyzed for mRNA levels of DGC components using quantitative real-time PCR (qRT-PCR) in 96-well optical reaction plates on a Step One Plus Real-time PCR system (Applied Biosystems), operated by Step One software version 2.3. Ct values were calculated for each gene and normalized to GAPDH, with three independent replicates per condition. Lastly, analysis of reaction melt curves was used to ensure quality control.

### Preparation of protein lysates from cells and tissue

Oligodendroglia were lysed in 20 mM Tris pH 7.4, 1% sodium dodecyl sulfate and a 1:50 dilution of protease (Set III, Calbiochem, CA, USA) and phosphatase inhibitor cocktails (Set II, Calbiochem, CA, USA). Cell lysates were scraped, transferred to microfuge tubes and heated at 95 °C for 15 min. For tissue, dissected *mdx* cerebral cortices, cerebellum, olfactory bulb and spinal cord at postnatal days 8, 14, 21, 6 weeks and 2 months were lysed in 20 mM Tris pH 7.4, 1% sodium dodecyl sulfate and a 1:50 dilution of protease (Set III, Calbiochem, CA, USA) and phosphatase inhibitor cocktails (Set II, Calbiochem, CA, USA). 200 mg of each tissue was suspended in 1 mL of lysis buffer, minced using a bio-vortexer, and heated at 95 °C for 15 min. Cell and tissue lysate protein concentrations were obtained using a detergent compatible protein assay kit (Bio-Rad, CA, USA). Lastly, cell and tissue lysates were mixed with Laemmli solubilizing buffer (LSB) sample buffer (NuPAGE; Invitrogen, CA, USA) containing 12% β-mercaptoethanol (Sigma-Aldrich, MO, USA), and heated again for another 5 min prior to −80 °C storage.

### Western blots

Sodium dodecyl sulfate polyacrylamide gel electrophoresis (SDS-PAGE) was used for protein separation. Acrylamide gels were either 0.75 or 1.5 mm thick and ranged from 7.5 to 15% (7.5, 10, 12, 15%), depending on the protein of interest. Protein gels were blotted onto 0.45 nitrocellulose membranes and blocked in 4% bovine serum albumin, suspended in Tris buffered saline with 0.1% Tween-20 (TBS-T), for 45 min. Membranes were incubated and rocked overnight at 4 °C in primary antibodies diluted in the BSA/TBS-T blocking solution. Following primary antibody incubations, membranes were washed three times with TBS-T, and unless otherwise indicated, incubated at room temperature for 1 h with LI-COR infrared-coupled (IR-800 or 700) secondary antibodies, suspended in the BSA blocking solution. After incubation with secondary antibodies, membranes were washed again three times in TBS-T. Detection of immune-reactive proteins was performed using LI-COR Odyssey detection Software and protein band intensity were normalized to loading controls (either β-actin or p115).

### Cell fractionation

Oligodendrocytes were detached using trypsinization then resuspended in TD1 buffer (135 mM NaCl, 5 mM KCl, 25 mM Tris–HCL, pH 7.6) and split into two fractions, A & B. Fraction A was dedicated to whole cell lysis and Fraction B to cell fractionation. Fraction A was centrifuged at low speed (3000 rpm) for 2 min then TD1 buffer was removed and replaced with a lysis buffer (consisting of 1% SDS, 20 mM Tris pH 7.4, protease and phosphatase inhibitor cocktails), prior to heating at 95 °C for 15 min. Whole cell lysates were then mixed with Laemmli solubilizing buffer (LSB) sample buffer (NuPAGE; Invitrogen, CA, USA) containing 12% β-mercaptoethanol (Sigma, MO, USA), and heated again at 95 °C for 5 min prior to −80 °C storage. Fraction B was centrifuged at 3000 rpm for 2 min to remove TD1 and add an ice-cold lysis resuspension buffer consisting of 10 mM NaCl, 1.5 mM CaCl_2_, 10 mM Tris–HCL, pH 7.5, both protease and phosphatase inhibitor cocktails, and 1% Triton X-100 in PBS. Cells were then centrifuged at 14,000 rpm for 10 min in 4 °C to separate cytoplasmic (supernatant) and nuclear (pellet) fractions. The nuclear pellet was washed three times in the lysis resuspension buffer, prior to sonication (Branson Digital Sonifier, model 450). Lastly, all the samples were mixed with Laemmli solubilizing buffer (LSB) sample buffer (NuPAGE; Invitrogen, CA, USA) containing 12% β-mercaptoethanol (Sigma, MO, USA), and heated at 95 °C for 10 min prior to −80 °C storage.

### siRNA transfection

To target dystrophin a pool of siRNA duplexes specific for the 3′ region (common to all isoforms) of rat dystrophin mRNA were used. This siGENOME Rat DMD siRNA smart pool (GE Healthcare Dharmacon, IL, USA) consisted of D-101558-13 (Target-GAUUAGACAGUAAGCGUUU), D-101558-14 (Target-CCUCAAAGGCCACGAGACC), D-101558-15 (Target-GCGCCAACACAAAGGACGU) and D-101558-16 (Target-CUUUAUAUGGAACGCAUUU). A pool of non-targeting siRNA (siCONTROL) were used as a control. Primary rat OPCs were transfected by electroporation using a Nucleofector II electroporation system (Amaxa Biosystems, Germany) and an Amaxa Basic Glial Cells Nucleofector Kit (Lonza, MD, USA), as done routinely in the Colognato lab, where previous work with this method had demonstrated a delivery efficiency of fluorescent siGLO siRNA (Dharmacon) to be approximately 90% [23]. Transfected OPCs were seeded into PDL-coated 35 mm tissue culture treated dishes or 8-well glass chamber slides (NalgeNunc International, NY, USA) overnight and incubated at 37 °C, 7.5% CO_2_ in proliferation (Sato medium with 10 ng/mL of both platelet-derived growth factor and basic fibroblast growth factor) media for 1 day, to ensure adequate time for knockdown. At 24 h post-transfection cells were switched to differentiation medium (Sato’s medium with 0.5% FBS) [31]. Cells were then monitored for up to 8 days, using immunohistochemistry and western blotting to assess oligodendrocyte differentiation.

### Mice and genotyping

Hemizygous males (x^MDX^ y; strain C57BL/10ScSn-Dmd-Mdx/J) and homozygous females (x^MDX^ x^MDX^; strain C57BL/10ScSn-Dmd-Mdx/J) pair were purchased from Jackson Laboratory. The homozygous females (x^MDX^ x^MDX^) were crossed with C57BL/6J (x^WT^ y) males to generate litters with heterozygous females (x^MDX^ x^WT^; phenotypically wildtype) and mutant males (x^MDX^ y). Heterozygous females served as controls to their aged-matched mutant male siblings (see Fig. 2a). Genotyping was performed on DNA obtained from mouse tails using a primer competition PCR as described in Shin et al. [30] (see Additional file 1: Fig. S1). Primers used for genotyping were *mdx* common forward (DL 1577), wildtype reverse (DL 1509) and mutant reverse (DL 1573) [30]. All mouse experiments were in compliance with the National Institutes of Health Guide for the Care and Use of Laboratory Animals and approved by the Stony Brook University’s Institutional Animal Care and Use Committee.

### Fluorescent immunohistochemistry using PFA-fixed sections

For *mdx* mice younger than postnatal day 14, *mdx* cerebral cortices were dissected and immersed in 4% paraformaldehyde (PFA) overnight at 4 °C. For *mdx* mice older than postnatal day 14, the mice were anesthetized using 2.5% avertin (diluted in PBS), followed by a 5 min 1× PBS intracardial perfusion to remove residual blood in the cerebral vasculature and a 25 min intracardial perfusion with 4% PFA. After perfusion, cortices were post-fixed in 4% PFA overnight at 4 °C. After overnight fixation, cerebral cortices were equilibrated in 30% sucrose at 4 °C before slow freezing in Tissue-Tek OCT compound (Sakura Finetek, CA, USA) using dry ice combined with 2-methylbutane (Fisher Chemical, NJ, USA). Frozen tissues underwent coronal sectioning via cryostat and were stored at −80 °C until immunohistochemistry could be performed. For fluorescent immunohistochemistry, sections were post-fixed on the slide as follows: 15 min ice-cold 4% PFA, followed by 25 min 95:5 ice-cold Ethanol: Acetic acid for MBP, 10 min ice-cold 100% methanol for Olig2/CC1, and 10 min ice-cold 95:5 Ethanol: Acetic acid for PDGFRα/ki67 and CC1/ENPP6. With the exception of sections destined for MBP detection, all other sections then underwent three washes with 1× PBS, a 30 min incubation at 95 °C in an Antigen Retrieval Solution, pH 6 (Dako Cytomation, Agilent, CA, USA), then a 15 min cool down and another three washes with 1× PBS. Sections were subsequently incubated in blocking solution at room temperature, consisting of 1% BSA, 1× PBS and 0.1% Triton X-100, for 45 min. Sections were subsequently washed 3 times in 1× PBS for prior to overnight incubation with primary antibodies in blocking solution at 4 °C. The following day, sections were washed 3 times in 1× PBS and incubated with secondary antibodies in blocking solution for 1 h, followed three washes in 1× PBS, a 10 min incubation with 4′,6-diamidino-2-phenylindole (DAPI; 1:1000 in 1× PBS) at room temperature to label cell nuclei and a final 1× PBS wash before mounting in Slow Fade Gold (Life Technologies, Grand Island, NY, USA).

#### TUNEL

 Sections were first treated with primary and secondary antibodies as described above and washed 3 times in 1× PBS. Next, sections were treated with freshly made cold permeabilization solution (0.1% Triton X-100, 0.1% sodium citrate) for 2 min, and washed three times in 1× PBS. Each section was then incubated with a mixture of 5 μL enzyme solution and 45 μL TUNEL-label solution mixture (Roche, In situ cell death detection fluorescein kit). Sections were incubated for 60 min at 37 °C in a humidified chamber. Lastly, TUNEL-labeled sections underwent three 1× PBS washes, a 10 min incubation with 4′,6-diamidino-2-phenylindole (DAPI; 1:1000 in 1× PBS) at room temperature and a final 1× PBS wash before mounting in Slow Fade Gold (Life Technologies, Grand Island, NY, USA).

### Fluorescent immunocytochemistry

Oligodendrocytes cultured on PDL-coated glass coverslips or chamber slides were initially fixed in 4% paraformaldehyde (PFA) for 10 min, then additionally treated as follows: for MBP, CNP, utrophin detection, 7.5 min in ice-cold 4% PFA; for β-dystroglycan detection, 7.5 min in ice-cold 100% methanol; for dystrophin detection, 7.5 min in ice-cold 1:1 methanol: acetone. Cells were washed in 1× PBS then incubated at room temperature in blocking solution (1% BSA, 1× PBS and 0.1% Triton X-100) for 45 min. Cells were then incubated at 4 °C overnight with primary antibodies in blocking solution. The following day, cells were washed 3 times in 1× PBS, then incubated with secondary antibodies in blocking solution for 1 h, followed by three washed in 1× PBS, a 10 min incubation with 4′,6-diamidino-2-phenylindole (DAPI; 1:1000 in 1× PBS) to label cell nuclei, and a final 1× PBS wash before mounting in Slow Fade Gold (Life Technologies, Grand Island, NY, USA). α-dystroglycan detection required labeling of live oligodendrocytes as follows. First, differentiation medium was removed and cells were incubated at room temperature with DMEM plus 10% FBS containing anti-α-dystroglycan (IIH6) for 20 min. Next, cells were washed 3 times with DMEM plus 10% FBS, then fixed in ice-cold 100% methanol for 7.5 min, followed by another 3 washes in 1× PBS. Cells were subsequently incubated with FITC-conjugated goat anti-mouse IgM for 1 h, washed three times with 1× PBS, incubated with DAPI for 10 min, then mounted with slow fade gold.

### Microscopy and image acquisition

Oligodendrocyte α- and β-dystroglycan images were obtained using a zeiss axiocam MRM digital camera and Zeiss Axioplan inverted epifluorescence microscope using 20× and 40× objectives, operating under the control of Axiovision software (Rel. 4.8; Carl Zeiss, Oberkocken, Germany). Oligodendrocyte dystrophin and MBP images were obtained using a Leica SP5 confocal microscope using 20× and 40× objectives, operating under Leica Application Suite AF software. The oligodendrocyte utrophin and all brain section images were obtained using a Leica TCS SP8 X Confocal Microscope using 20×, 40×, and 63× oil immersion objections, operating under Leica Application Suite X (LAS X) software.

### Statistical analysis

SigmaPlot11 and Excel were used to perform Student’s two-tailed, paired t-tests and ANOVAs (one-way ANOVA, Student-Newman–Keuls). Compared groups were deemed significantly different if p values had a 0.05 confidence value (* represent p values <0.05, ** represent p values <0.01, and *** represent p values <0.001). The graphs displayed indicate mean values and error bars indicate standard error of the mean (SEM).

## Results

### Dystrophin glycoprotein complex (DGC) members are expressed in oligodendroglia

**Fig. 1:**
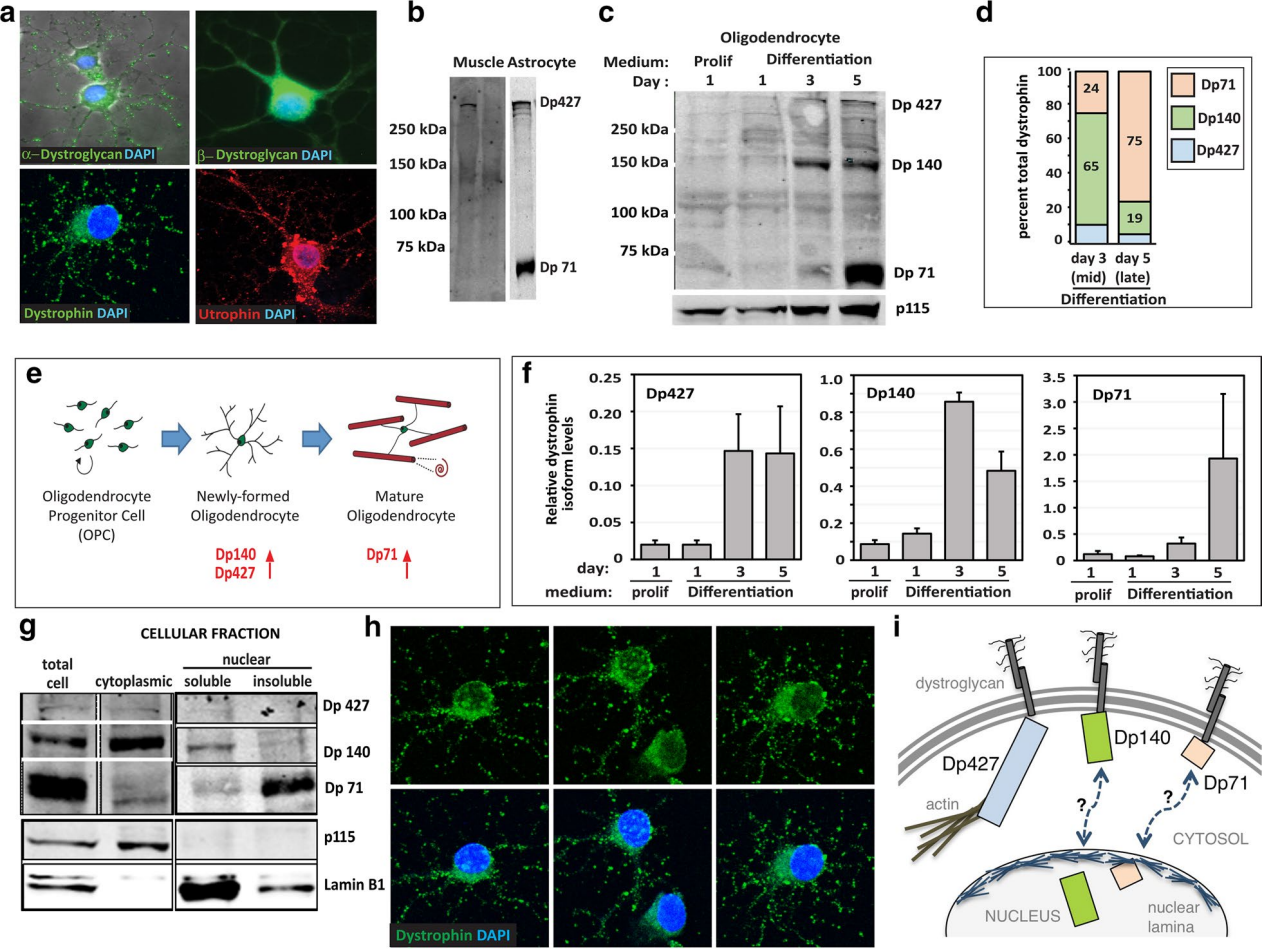
Oligodendrocytes express dystrophin glycoprotein complex (DGC) components and multiple dystrophin isoforms. **a** Immunocytochemistry on rat oligodendrocytes following 3 days of differentiation to detect selected DGC components (α-dystroglycan, β-dystroglycan, dystrophin, and utrophin), **b** western blot analysis dystrophin protein in skeletal muscle lysates (*mdx*/+ and *mdx*) and wildtype astrocyte lysates, **c** OPCs were cultured either in proliferation (prolif) or differentiation medium, then analyzed at days 1, 3 and 5 by western blot to detect dystrophin proteins. The positions of molecular weight standards are indicated. p115 immunoblots were used as loading controls. Three distinct dystrophin isoforms were detected: Dp427, Dp140, and Dp71, **d** the percentage of each dystrophin isoform, relative to the total dystrophin expressed at each time point, was calculated for oligodendrocytes at days 3 and 5, **e** schematic of oligodendrocyte maturation indicating at which maturation state particular dystrophin isoforms are upregulated, **f** the relative levels of dystrophin isoforms in OPCs (prolif) or oligodendrocytes at days 1, 3, and 5 in differentiating-promoting medium, **g** oligodendrocytes differentiated for 5 days (mature stage) were lysed and subjected to subcellular fractionation, followed by western blot analysis to detect dystrophin isoform proteins within the total cell, cytoplasmic, and nuclear fractions (soluble and insoluble). Dp427 and Dp140 were found in the cytoplasmic fraction, Dp140 was found in the soluble nuclear fraction, and Dp71 was found in the insoluble nuclear fraction. Cellular fractionation efficacy was verified with blotting for p115, which recognizes a vesicle docking protein that is cytoplasmic, and blotting for Lamin B1, a nuclear envelope protein, **h** confocal images of mature oligodendrocytes following dystrophin immunocytochemistry (*green*) reveal that dystrophin is distributed in the oligodendrocyte cell body, cell processes, and nucleus, **i** schematic depicting different pools of oligodendrocyte dystrophins

A canonical DGC component, dystroglycan, is expressed in developing oligodendrocytes [23], where it was found to be necessary for timely oligodendrocyte differentiation and oligodendrocyte process branching [23, 24, 26]. We therefore hypothesized that oligodendrocytes might also express the dystroglycan binding partner, dystrophin and an associated DGC. We isolated mRNA from primary rat oligodendroglial cultures grown on PDL in differentiation-promoting medium for 1, 3, or 5 days, and assessed mRNA levels of the following established DGC components: dystrophin, dystroglycan, sarcoglycans (α, β, δ, ε, γ, ζ), dystrobrevins (α, β) and syntrophins (α1, β1, β2, γ1, γ2), utrophin (DRP1) and dystrophin-related protein 2 (DRP2). Using real time quantitative reverse transcriptase polymerase chain reaction (qRT-PCR), we detected the presence of all 17 putative DGC components (Table 1). Abundancy of cT (values relative to the housekeeping gene, GAPDH) ranged from 10^−4^ to 10^−2^, indicating that transcript levels vary considerably among DGC components. Some transcripts were present at very high levels (e.g., dystroglycan, sarcoglycan-β), while others were at moderate (e.g., dystrophin, sarcoglycan-α) or lower (e.g., syntrophin-β2, utrophin) levels (Table 1). We also observed a maturation state dependent regulation of transcript levels for many DGC genes, suggesting possible DGC remodeling during differentiation (Table 2). We were therefore able to group DGC components across the 5 day differentiation period as either remaining constant, increasing, or decreasing (Table 2, column heading “Day 1–5 pattern”). At day 1, oligodendroglia are mostly oligodendrocyte progenitor cells (OPCs) or very newly-formed immature oligodendrocytes, but by day 5 most oligodendroglia have transitioned into mature oligodendrocyte capable of producing myelin proteins (see schematic, Fig. 1e).

**Table 1:** mRNA levels of dystrophin-glycoprotein complex (DGC) components in oligodendrocytes

(relative to GAPDH, n = 3)		
~10^−4^	~10^−3^	~10^−2^
Dystrobrevin-Beta	Dystrobrevin-Alpha	Dystroglycan
Sarcoglycan-Delta	Dystrophin related protein 2 (DRP2)	Sarcoglycan-Beta
Sarcoglycan-Zeta	Dystrophin	
Syntrophin-Alpha1	Sarcoglycan-Alpha	
Syntrophin-Beta2	Sarcoglycan-Epsilon	
Syntrophin-Gamma2	Syntrophin-Beta1	
Utrophin	Syntrophin-Gamma1	
	Sarcoglycan-Gamma

**Table 2:** mRNA levels of DGC components in differentiating oligodendrocytes (relative to GAPDH, n = 3)

Target gene	Substrate	Differentiation media	Differentiation media	Differentiation media	
		Day 1 (10e−5)	Day 3 (10e−5)	Day 5 (10e−5)	Day 1–5 pattern
Dystrobrevin-Alpha	PDL	139 ± 10	340 ± 15	703 ± 19	Increase
	Lm-2	93 ± 6	305 ± 08	826 ± 54	Increase
Dystrobrevin-Beta	PDL	54 ± 3	31 ± 2	32 ± 1	No change
	Lm-2	58 ± 7	22 ± 2	22 ± 2	No change
Dystroglycan	PDL	3235 ± 217	3387 ± 168	5543 ± 245	No change
	Lm-2	3096 ± 406	3225 ± 455	3814 ± 225	No change
Dystrophin	PDL	205 ± 0.5	360 ± 19	562 ± 34	No change
	Lm-2	216 ± 20	240 ± 4	410 ± 47	No change
Dystrophin related protein 2 (DRP2)	PDL	106 ± 6	85 ± 5	110 ± 10	No change
	Lm-2	140 ± 7	51 ± 2	49 ± 4***	Decrease
Sarcoglycan-Alpha	PDL	218 ± 27	328 ± 21	585 ± 13	Increase
	Lm-2	178 ± 2	243 ± 35	359 ± 18***	No change
Sarcoglycan-Beta	PDL	1976 ± 70	2091 ± 75	3215 ± 49	Increase
	Lm-2	1776 ± 151	1614 ± 134	2048 ± 61***	No change
Sarcoglycan-Delta	PDL	53 ± 3	26 ± 0.4	27 ± 0.5	Decrease
	Lm-2	41 ± 2	24 ± 0.6	32 ± 2	No change
Sarcoglycan-Epsilon	PDL	175 ± 6	10 ± 2	139 ± 0.9	No change
	Lm-2	150 ± 6	99 ± 1	156 ± 6	No change
Sarcoglycan-Gamma	PDL	27 ± 4	42 ± 4	73 ± 3	Increase
	Lm-2	25 ± 0.5	19 ± 1	41 ± 1***	No change
Sarcoglycan-Zeta	PDL	57 ± 4	43 ± 4	38 ± 3	No change
	Lm-2	64 ± 6	28 ± 0.4	22 ± 2	No change
Syntrophin-Alpha1	PDL	44 ± 3	64 ± 3	100 ± 4	No change (p = 0.079)
	Lm-2	33 ± 5	48 ± 1	123 ± 9	Increase
Syntrophin-Beta1	PDL	251 ± 14	111 ± 6	132 ± 7	Decrease
	Lm-2	252 ± 9	121 ± 10	128 ± 9	Decrease
Syntrophin-Beta2	PDL	48 ± 6	41 ± 1	63 ± 5	No change
	Lm-2	16 ± 0.6	9 ± 0.3	10 ± 0.1	No change
Syntrophin-Gamma1	PDL	232 ± 21	106 ± 3	159 ± 5	No change
	Lm-2	115 ± 6	108 ± 4	141 ± 6	No change
Syntrophin-Gamma2	PDL	43 ± 2	18 ± 0.6	24 ± 2	No change
	Lm-2	24 ± 3	16 ± 0.6	28 ± 0.9	No change
Utrophin	PDL	16 ± 0.4	12 ± 0.1	15 ± 0.8	No change
	Lm-2	16 ± 0.6	9 ± 0.3	10 ± 0.1	Decrease

*** Significant differences in DGC component mRNA levels when comparing cells grown on PDL to those on Laminin

To determine if DGC transcript levels change in response to the ECM protein laminin, a dystroglycan ligand, oligodendrocytes plated on laminin were differentiated in parallel to those differentiated on PDL (an adhesive but non-ECM substrate). A subset of DGC transcripts appeared to be regulated by substrate, e.g., increasing or decreasing on laminin while remaining constant on PDL, or vice versa (Table 2). Since laminin is transiently found associated with axons in nascent white matter tracts during postnatal myelination [32], alterations in DGC composition in response to laminin could be a useful mechanism to modulate oligodendroglia following oligodendroglial-axon interactions.

### Oligodendrocytes express three dystrophin isoforms that are developmentally regulated

We next confirmed the presence of dystrophin in oligodendrocytes by immunocytochemistry (Fig. 1a, h), finding dystrophin protein in the cell body as well as in the multiple fine processes found in maturing oligodendroglia. This widespread distribution mirrored that of dystroglycan, as did the dystrophin homologue utrophin (Fig. 1a). However, as the dystrophin antibody recognized the C-terminal region of dystrophin that is common among the major dystrophin isoforms, we next sought to evaluate protein lysates by western blot to determine which dystrophin isoform(s) were present in oligodendrocytes. To assess the specificity of the dystrophin antibody for western blot, we assessed protein lysates from *mdx* (or littermate control) skeletal muscle, where we observed the largest isoform of dystrophin, Dp427, solely in control lysates (Fig. 1b). This same dystrophin antibody, applied to blots of wildtype astrocyte lysates, detected both Dp427 as well as the smaller Dp71 isoform, the isoforms that have been previously identified in astrocytes [33]. Next, we assessed oligodendroglial lysates at various stages in development: as oligodendrocyte progenitor cells (OPC), and after 1, 3, or 5 days in differentiation promoting medium (Fig. 1c). We found oligodendrocytes to possess three of the five major dystrophin isoforms: Dp427 (the classic, full length dystrophin) and two shorter isoforms, Dp140 and Dp71. Intriguingly, while all three isoforms were barely visible in OPCs, they were upregulated as OPCs differentiated into oligodendrocytes (Fig. 1c, f), suggesting a role in the oligodendroglial differentiation process. By analyzing the fraction of each isoform out of “total dystrophin”, we determined that Dp427 and Dp140 were the predominant dystrophin isoforms in newly-differentiated, immature oligodendrocytes (day 3), while Dp71 was the most prominent isoform in myelination-competent mature oligodendrocytes (day 5) (Fig. 1d, e). In summary, oligodendrocytes have multiple dystrophin isoforms whose expression appears to be developmentally regulated, providing insight into potential dystrophin functions during myelination.

In other cell types, both dystroglycan and dystrophin have been found to translocate to the nucleus [34–36]. In those studies both dystroglycan and dystrophin isoform Dp71 were found in association with nuclear lamina proteins however the function of dystroglycan and Dp71 in the nucleus remains unclear [34–36]. In immunocytochemistry images obtained using confocal microscopy, we noted substantial dystrophin immunoreactivity in the oligodendrocyte nucleus (see Fig. 1h, where DAPI is removed to better visualize dystrophin immunoreactivity within the nucleus). To determine if dystrophin was indeed found in the oligodendrocyte nucleus, we performed cell fractionation on protein lysates obtained from mature oligodendrocytes (day 5 differentiation; Fig. 1g). Dp427 was detected solely in the cytoplasmic fraction, while both Dp140 and Dp71 were present in nuclear fractions, with Dp71 being particularly enriched in the more detergent-resistant “nuclear insoluble” fraction that is likely nuclear lamina associated material; see “Methods”. Together, these results indicate that different dystrophin isoforms may adopt different cellular locations during oligodendrocyte development, indicating a possible diversity in the functions of the individual isoforms.

### Developmental myelination deficits in *mdx* mice, a mouse model of Duchenne muscular dystrophy

**Fig. 2:**
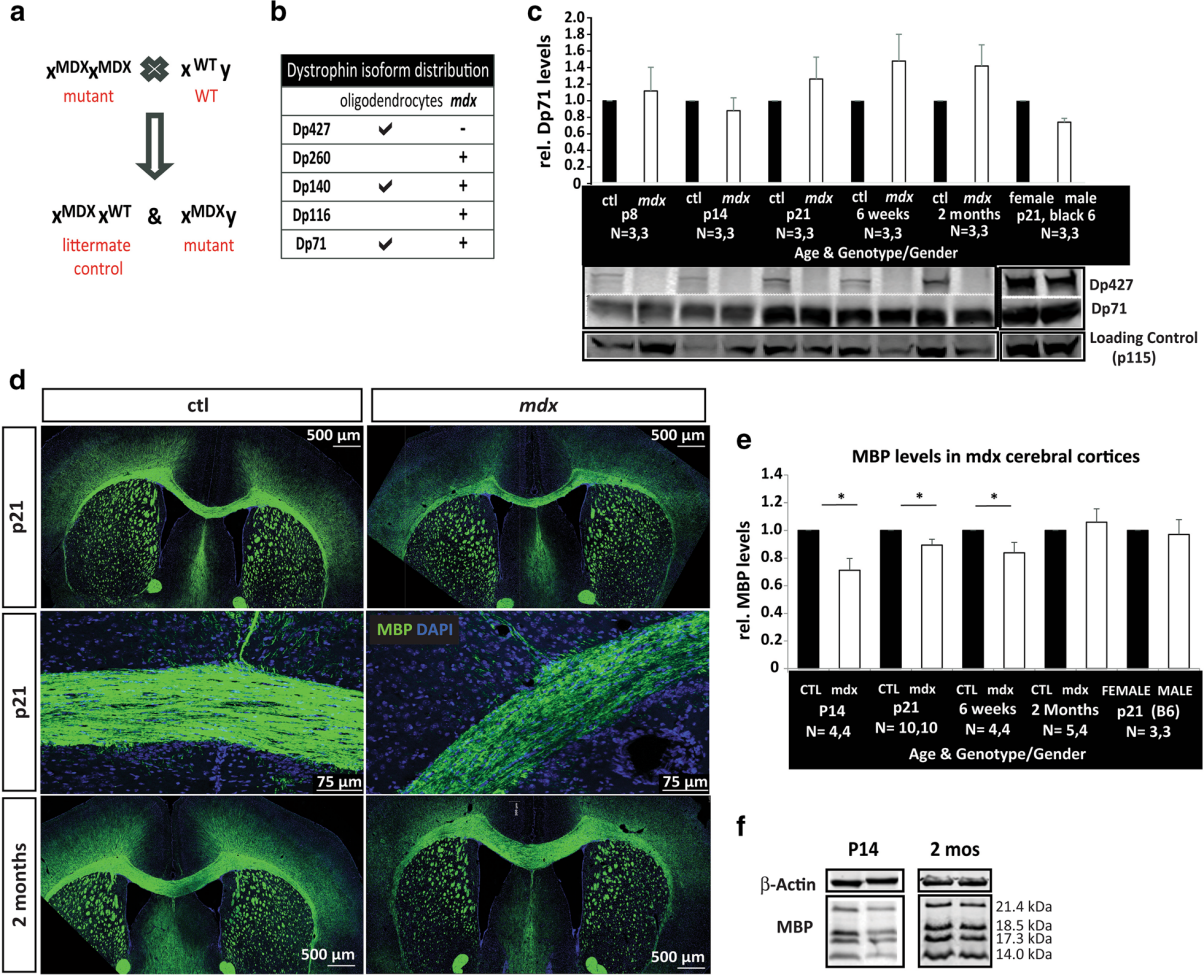
Myelination is delayed during postnatal brain development in the *mdx* mouse model of DMD. **a** Breeding strategy to generate litters with *mdx* males and heterozygous *mdx*/+ females (controls), **b** dystrophin isoform expression in oligodendrocytes and *mdx* mice, **c** western blot analysis of dystrophin protein content in lysates obtained from *mdx* and control cerebral cortices at postnatal day 8 (p8), p14, p21, 6 weeks, and 2 months. Comparable levels of Dp71 are found in both *mdx* and control lysates, while Dp427 is absent from *mdx* lysates. Cortical lysates from wildtype male and female mice (B6, Black 6) reveals that Dp427 and Dp71 levels are similar in males and females. Representative western blots are shown, including those for p115 as a loading control, **d** MBP immunohistochemistry of *mdx* and control coronal floating brain sections. Tiled confocal microscopy images reveal less MBP immunoreactivity in *mdx* cerebral cortices at postnatal day 21 (p21) but not at 2 months, **e** quantification of mean MBP densitometries from western blots comparing protein lysates from cerebral cortices of control and *mdx* mice at p14, p21, 6wks and 2 months. In addition, cortical lysates from wildtype mice (B6, Black 6) were analyzed to ensure that MBP levels did not differ by gender. Immunoblots to detect β-actin were used as loading controls. MBP levels in *mdx* cerebral cortices were decreased at p14, p21, and 6 weeks but were normal by 2 months (*p < 0.05), **f** representative western blots of cortical lysates from postnatal day (p14) and 2 month old (2 mos) *mdx* and control mice

Studies on laminin- and dystroglycan-deficient mice suggest that laminin-dystroglycan interactions are required for normal brain myelination [20, 21, 26]. To determine which aspects of oligodendrocyte development and myelination might require the presence of dystrophin, an intracellular binding partner for dystroglycan, we evaluated postnatal brain development in *mdx* mice, a mouse model of Duchenne muscular dystrophy (DMD). *Mdx* mice lack Dp427, but retain expression of the shorter dystrophin isoforms (see Fig. 2b), and have aberrant brain function, including a leaky blood–brain-barrier and behavioral deficits [18, 19, 37–39]. Homozygous females (x^MDX^ x^MDX^) were crossed with wildtype C57BL/6J (x^WT^ y) males to generate litters consisting of heterozygous females (x^MDX^ x^WT^; phenotypically wildtype) and mutant males (x^MDX^y). These heterozygous female littermates served as controls for comparison to aged-matched mutant male siblings (Fig. 2a). To confirm the expected *mdx* dystrophin expression profile, we assessed levels of Dp427 and Dp71 protein in *mdx* mutant and littermate control cerebral cortices and found that, as expected, Dp427 was absent but Dp71 levels remained high, i.e., similar to that in control littermates (Fig. 2c).

To assess developmental myelination, immunohistochemistry and confocal microscopy were used to generate tiled images of myelin basic protein (MBP) immunoreactivity in the corpus callosum and surrounding regions in coronal sections from mice of both genotypes at postnatal day 21 (p21) and 2 months of age (Fig. 2d). We observed decreased MBP immunoreactivity in p21, but not 2 month old, brain sections, indicative of a possible myelination delay. We next used western blot analysis to evaluate levels of MBP protein in the cerebral cortex at developmental time points spanning onset, peak (~p21), and completion of myelination: p8, p14, p21, 6 weeks and 2 months. As expected, MBP was barely detectable at p8, so MBP analysis was only performed at p14 and onwards (Fig. 2e). Relative to control littermates, there was a decrease in total MBP protein levels (defined as the sum of 14.0, 17.3, 18.5, and 21.4 kDa MBP isoforms) in the *mdx* mutant cerebral cortices than controls at p14, p21 and 6 weeks (Fig. 2e). However, by 2 months of age, *mdx* mice had normal MBP levels (Fig. 2e), confirming myelination was developmentally delayed but not permanently attenuated. In addition, cerebral cortices were analyzed by western blot analysis for levels of 2′,3′-cyclic nucleotide 3′-phosphodiesterase (CNP) levels, an oligodendrocyte-specific protein found in both immature and mature oligodendrocytes. Interestingly, *mdx* CNP levels were normal at all time points with the exception of 2 months, where *mdx* cortices had slightly more CNP than normal (Additional file 2: Fig. S2), indicating that oligodendroglial maturation deficits occurred after oligodendrocytes reached the CNP-positive stage, i.e., during the later stages of oligodendrocyte maturation. Finally, we also assessed myelination in lysates from additional brain structures, the cerebellum and spinal cord. MBP levels were mostly similar in these structures however small but significantly lower levels of MBP were found at p21 in spinal cord lysates and p14 in cerebellum lysates (Additional file 3: Fig. S3) from *mdx* mice. However, we found no significant differences in CNP levels of spinal cord and cerebellum between *mdx* and control littermates at any developmental time point (data not shown). Together these data indicate that the central nervous system of *mdx* mice has delayed myelination that is most pronounced in the cerebral cortex.

### The *mdx* corpus callosum has abnormally high densities of immature oligodendrocyte progenitor cells

**Fig. 3:**
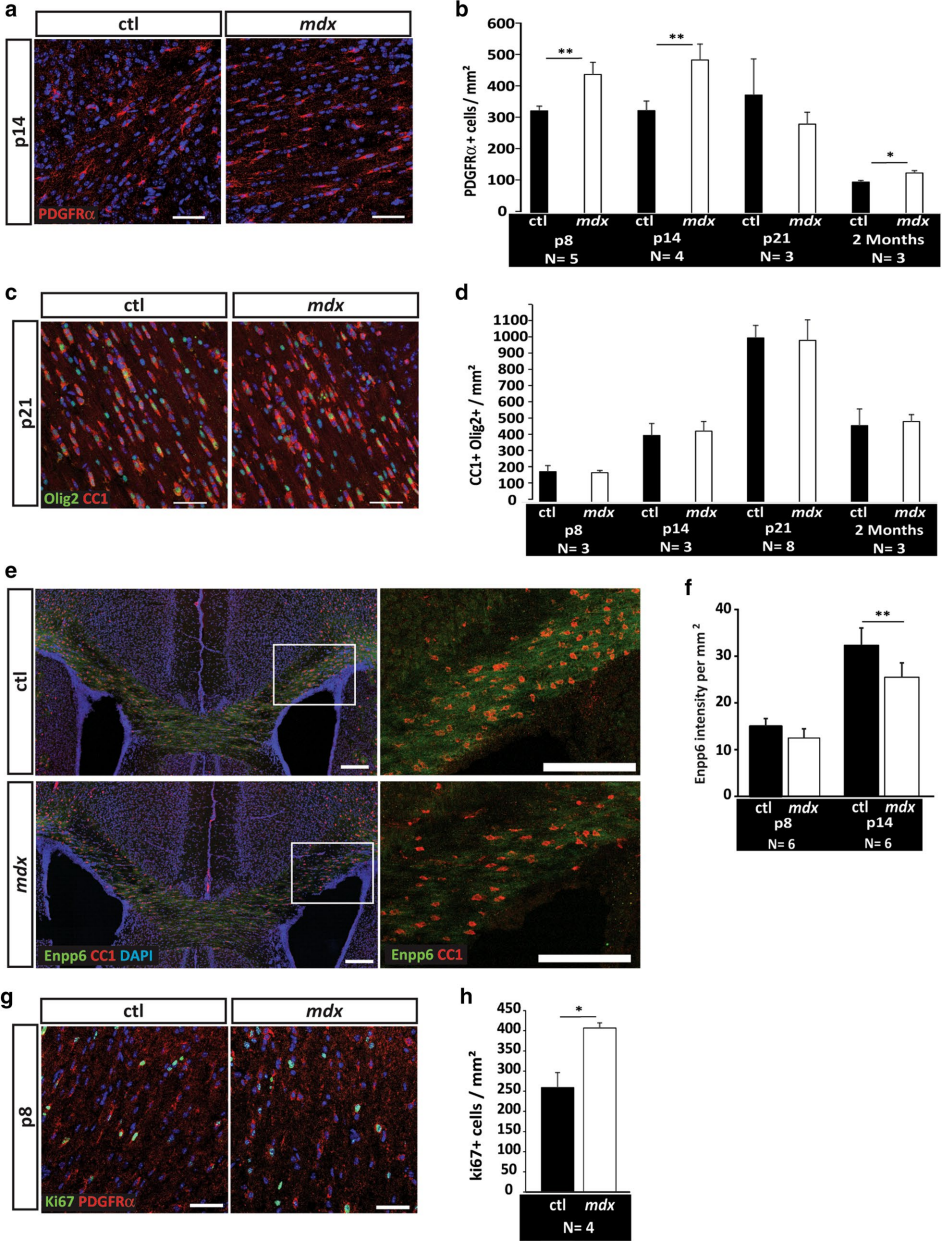
Abnormal densities of oligodendrocyte progenitor cells are accompanied by normal oligodendrocyte densities in the developing *mdx* corpus callosum. **a** Oligodendrocyte progenitor cells (OPCs) in the developing corpus callosum white matter tract were visualized using PDGFRα immunohistochemistry. Representative confocal microscopy images of postnatal day 14 (p14) *mdx* and control callosa are shown, **b** quantification of mean PDGFRα+ cells (OPCs) per mm^2^ in confocal images from p8, p14, p21, and 2 months corpus callosum. Abnormally high OPC densities were observed in *mdx* mice relative to that in controls. ***p < 0.01, **c** oligodendrocytes in the developing corpus callosum white matter tract were visualized using CC1 (oligodendrocyte) and olig2 (oligodendrocyte lineage) immunohistochemistry. Representative confocal microscopy images of p21 *mdx* and control callosa are shown, **d** quantification of mean CC1+ Olig2+ cells (oligodendrocytes) per mm^2^ in confocal images from p8, p14, p21, and 2 months corpus callosum. Oligodendrocyte densities were similar in *mdx* and control mice, **e** Enpp6 immunohistochemistry of *mdx* and control coronal floating brain sections at postnatal day 14 (p14) and p8. Tiled confocal microscopy images reveal less Enpp6 immunoreactivity in *mdx* cerebral cortices, **f** quantification of Enppp6 intensity per mm^2^ in confocal images from p8 and p14 corpus callosum. Abnormally low Enppp6 intensity was observed in *mdx* mice relative to that in control mice. **p < 0.01, **g** proliferating OPCs in the developing corpus callosum white matter tract were visualized using PDGFRα and Ki67 immunohistochemistry. Representative confocal microscopy images of p8 *mdx* and control callosa are shown, **h** quantification of mean Ki67+ cells per mm^2^ in confocal images from p8 *mdx* and control corpus callosum. Ki67+ cell densities were significantly increased in *mdx* callosa relative to that in controls (*p < 0.05). *Scale bars* 50 microns

To determine if the myelination delay resulted from a lack of oligodendrocytes, we used immunohistochemistry to assess oligodendrocyte progenitor cell (PDGFRα-positive) and oligodendrocyte (CC1-positive) densities within the corpus callosum (Fig. 3a, c). Interestingly, at postnatal days 8 and 14, we observed an increase in oligodendrocyte progenitor cell (OPC) densities in the *mdx* corpus callosum compared to that in controls (Fig. 3a, b). Surprisingly, despite the abnormally high OPC densities, we observed no significant difference in oligodendrocyte densities at all developmental time points analyzed (Fig. 3d), indicating that the ability of OPCs to transition into oligodendrocytes was not impaired. We next evaluated Enpp6, an enzyme that is expressed in new, but not mature, oligodendrocytes, and found significantly less Enpp6 immunoreactivity in the corpus callosum of *mdx* mice at postnatal day 14 (Fig. 3e, f). Less Ennp6 immunoreactivity may reflect a deficit of immature oligodendrocytes or a decrease in cell area occupied by immature oligodendrocytes (which could occur if nascent axon contact/wrapping stages were delayed). Normal oligodendrocyte densities coupled with a deficit in both MBP and Enpp6 immunoreactivity indicates that individual *mdx* oligodendrocytes may fail to adequately mature. To explore the cause of increased OPC densities, we evaluated the density of proliferative (Ki67-positive) cells in the corpus callosum and found a significantly higher number of Ki67+ proliferating progenitor cells in the *mdx* callosum compared to that in control littermates (Fig. 3g, h). Finally, to determine whether increased levels of cell death could account for why normal oligodendrocyte densities arise despite higher OPCs densities, TUNEL in conjunction with CC1 and Enpp6 immunohistochemistry was performed on coronal sections from both *mdx* and control littermate brains at postnatal days 8 and 14 (Additional file 4: Figure S4). While *mdx* callosa had on average slightly more TUNEL+ cells in both time points, these changes were not statistically significant and therefore unlikely to be a contributing factor.

### The loss of all dystrophin isoforms in primary oligodendrocytes delays oligodendrocyte maturation

**Fig. 4:**
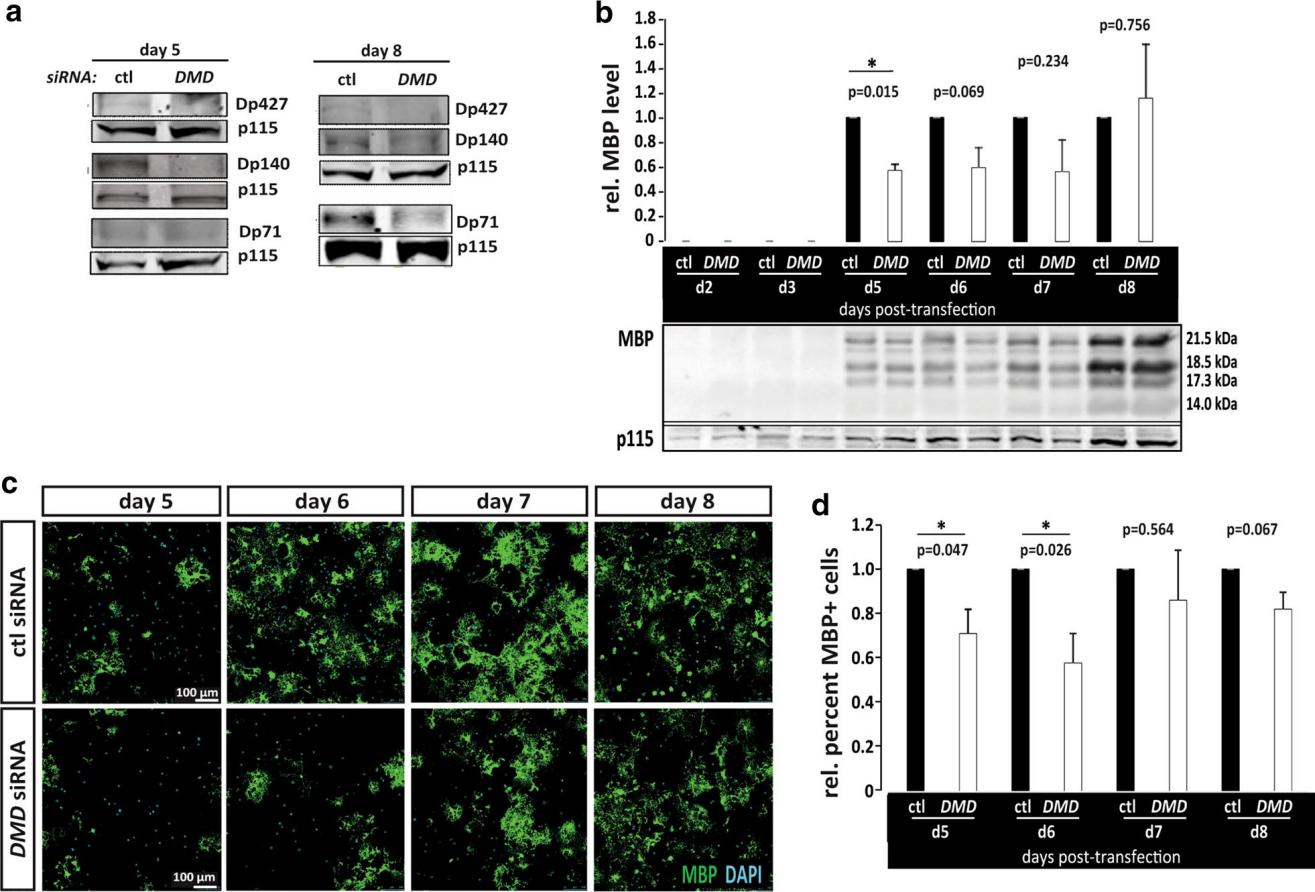
Dystrophin-deficient oligodendrocytes have maturation delays. **a** Western blot analysis of dystrophin proteins in oligodendrocyte lysates at day 5 and day 8 following transfection with dystrophin (DMD) or control (ctl) siRNA, **b** western blot analysis of total myelin basic protein (MBP) content in lysates obtained from differentiating oligodendrocytes at indicated days post-transfection with DMD or control siRNA. Representative western blots are shown, including those for p115 as a loading control. At day 5, dystrophin knockdown oligodendrocytes have significantly less MBP than control cells (p < 0.05), however MBP levels “catch up” by day 8, **c** oligodendrocytes were transfected with control (ctl) or dystrophin (DMD) siRNA and differentiated for the indicated times. Immunocytochemistry was then used to visualize MBP+ oligodendrocytes. Representative images are shown, **d** quantification of the mean relative percent MBP+ cells (out of total cells) in oligodendrocytes transfected with control (ctl) or dystrophin (DMD) siRNA at indicated time points. At days 5 and 6, there were significantly fewer MBP+ cells within the dystrophin-deficient oligodendrocytes (p < 0.05), however by day 8 dystrophin-deficient cells were similar to control cells

To learn whether myelination delays in *mdx* cerebral cortices were likely due to oligodendrocyte-intrinsic defects, we isolated primary oligodendrocytes (see “Methods”) and performed RNA interference targeting all dystrophin transcripts through their common 3′ region. First, we verified that the pool of 4 siRNA duplexes was able to substantially reduce all three oligodendroglial dystrophin isoforms (Dp427, Dp140, and Dp71), and that knockdown persisted throughout the timeframe of an oligodendrocyte maturation experiment, i.e., up to 8 days (Fig. 4a). We then monitored oligodendrocyte maturation by using western blots to analyze total MBP levels, relative to the level of p115 (loading control). We found that oligodendrocytes transfected with dystrophin siRNA had significantly less MBP at earlier stages of oligodendrocyte maturation (i.e., day 5), but by day 8 MBP levels were not significantly different than those in control transfected cultures (Fig. 4b). These data indicate that, similar to what was observed in vivo in *mdx* cerebral cortices, oligodendrocyte maturation is delayed by dystrophin loss. We next performed MBP immunohistochemistry to further assess oligodendrocyte maturation in dystrophin-deficient cells, where we quantified the percentage of cells that were MBP-positive at each differentiation time point (Fig. 4c). In dystrophin-deficient oligodendrocytes we found significantly fewer MBP+ cells at earlier stages (day 5 and 6 in differentiation medium) but similar densities of MBP+ cells at later stages (Fig. 4d). In addition, we assessed CNP levels in oligodendrocytes transfected with dystrophin or control siRNA and found no significant differences in CNP levels, at any time point (Additional file 5: Fig. S5), similar to what was observed in *mdx* cerebral cortices (Additional file 2: Fig. S2). Together, these data indicate that dystrophin-deficient oligodendrocytes have a cell-intrinsic deficit in maturation such that the expression of normal levels of myelin proteins is significantly delayed.

## Discussion

In the current study, we discovered that oligodendrocytes express the Dp427, Dp140 and Dp71 dystrophin isoforms, which are accompanied by a full complement of DGC members. We furthermore identified a developmental myelination delay in the cerebral cortices of the *mdx* mouse model of DMD, which lack Dp427, the dystrophin isoform that is critical in skeletal muscle and many other tissues. Finally, cultured primary oligodendrocytes were found to require dystrophin for appropriate and timely maturation, demonstrating that myelination deficits in *mdx* brains were likely due, at least in part, to oligodendrocyte-intrinsic requirements for dystrophin. Together, these findings indicate that inappropriate oligodendrocyte maturation and/or myelination delays have the potential to contribute to developmental neurological problems that are present in a large fraction of DMD patients [9].

In addition to identifying DGC components, we found that the DGC composition and dystrophin isoform expression within the DGC changes as oligodendrocytes move through the maturation process. DGC components in other cells have also been reported to undergo similar developmental changes, for example during adipogenesis, α-dystrobrevin expression increases developmentally and dystrophin isoform expression is altered in response to extracellular matrix [40]. In the current study, we found that several elements of the DGC were altered in response to laminin, a key ECM ligand for dystroglycan. In the brain, developing oligodendrocytes are thought to come into contact with laminins, which are only very transiently found along axon tracts such as the corpus callosum at the onset of myelination [32], raising the intriguing possibility that axon-associated laminins may serve as regulators of oligodendroglial DGC composition.

What are the functions of the oligodendroglial DGC? An emerging theme from studies in multiple cell types is that DGC composition and functions may be highly cell type-specific. Some of these functions include linking the extracellular matrix to the actin cytoskeleton [41], plasma membrane structural integrity or stability [42, 43], clustering of receptors, transporters, ion channels [44], and localizing signaling proteins to the plasma membrane [3, 43]. In addition to differential DGC repertoires, cell- and tissue-specific promoters drive expression of multiple dystrophin gene products in a cell-specific manner [9, 12]. In the current study, we revealed that oligodendrocytes express three different dystrophin isoforms, whose expression is developmentally regulated. In addition, both Dp140 and Dp71 could be found in the oligodendrocyte nucleus, while Dp427 was not. With the discovery that oligodendrocytes express multiple dystrophin isoforms, which have differences in their subcellular localization, one possibility is that oligodendrocytes possess dystrophin isoform-specific DGCs with distinct functions. Given that mature oligodendrocytes have Dp140 and Dp71 as the predominant dystrophin isoforms, and both of these isoforms lack the actin-binding N-terminal domain of Dp427, it is unlikely that dystrophin’s role in mature oligodendrocytes is as a “structural bridge” from ECM to cytoskeleton. However, Dp140 and Dp71 may influence nuclear DGC interactions, with the potential to influence DNA organization, DNA replication, and transcription, as has been previously suggested for Dp71 in HeLa, PC12 neuronal and C2C12 muscle cell lines [34–36]. However, since there is no known nuclear localization signal (NLS) contained within dystrophin, we can only speculate that dystrophin isoforms might access the nucleus through β-dystroglycan associations or by possessing a non-canonical, yet functional NLS. Furthermore, Dp140 and Dp71 may also influence dystroglycan function at the plasma membrane, as these dystrophin isoforms possess the shared C-terminal domain that permits dystroglycan-binding. For instance, dystrophin binding to dystroglycan influences the ability of Src family kinases to phosphorylate a tyrosine near dystroglycan’s C-terminus [45, 46], which in turn influences dystroglycan stability and turn-over [45].

Dp427 itself appears to be restricted to the plasma membrane and likely performs more traditional dystrophin roles such as integrating ECM and cytoskeletal responses. Dp427 is clearly important for oligodendrocyte development, as our in vivo analysis of *mdx* mice revealed that Dp427 deficiencies are detrimental to the myelination process. However given that *mdx* mice lack Dp427 dystrophin in *all* cells, it remains possible that dystrophin-deficiencies in other CNS cell types indirectly influence oligodendroglial function. For example, astrocytes express dystrophin, and astrocyte dysfunction is capable of disturbing myelination (Domingues et al. [47]). In addition, *mdx* mice have inefficiencies in nerve transmission in a subset of GABAergic interneurons (Früh et al. [48]). To address whether oligodendrocytes themselves are affected by dystrophin loss in the *mdx* brain, we used siRNA to knockdown all dystrophins in primary oligodendrocytes grown in isolation. Given that we found similar myelin protein deficits in dystrophin-deficient oligodendrocytes that we had observed in *mdx* mice, it is likely that *mdx* myelination deficits result, at least in part, due to dysfunctional oligodendrocytes. How might dystrophin regulate oligodendrocyte function? One possibility is that dystrophin acts in concert with dystroglycan, which has previously been shown to regulate oligodendrocyte development, both in vitro [23, 24] and more recently, in vivo [26]. Specifically, delayed myelination and inappropriately high densities of oligodendrocyte progenitor cells are found in dystroglycan conditional knockout mice, a phenomena suggestive of delayed differentiation similar to LAMA2−/− phenotypes [20, 21]. Dystroglycan loss-of-function also reduces the ability of cultured oligodendrocytes to develop complex process branches [23–25], a deficit that is predicted to impede oligodendroglia in making appropriate numbers of axon contacts, potentially causing myelination delays as additional oligodendrocytes would be needed to fulfill myelination needs (each oligodendrocyte myelinates up to 50 axons).

To explore the possibility that the *mdx* myelination deficit results from oligodendrocyte dysfunction, we hypothesized that there might be reductions in oligodendrocyte populations within the developing white matter tracts of corpus callosum of *mdx* mice compared to controls. Intriguingly, there were no abnormalities in the mature oligodendrocyte densities in *mdx* brains, in contrast to what was observed in laminin-deficient brains where oligodendrocyte densities were significantly reduced [22]. On the other hand, we did observe an increase in oligodendrocyte progenitor (OPC) densities in *mdx* brains, relative to those in controls. And, Enpp6 levels are reduced in *mdx* brains, indicating abnormalities in newly-formed oligodendrocytes. However, given the normal numbers of oligodendrocytes, the pipeline of newly-formed oligodendrocytes, while reduced, is likely to be sufficient, given the normal numbers of CC1+ oligodendrocytes. It should be noted that Enpp6 is found in the nascent myelin sheaths (Morita et al. [49]) and thus reduced Enpp6 immunoreactivity, in conjunction with normal densities of CC1+ oligodendrocytes, could reflect a deficit in oligodendrocyte process extension along axons during early stages of myelination.

We also explored whether cell proliferation could account for the increased progenitor numbers, and found significantly more proliferating cells in the *mdx* corpus callosum. This finding suggests that the loss of dystrophin leads to increased densities of proliferating OPCs, likely by enhancing proliferation in the OPCs themselves. However, since oligodendrocyte progenitors are known to divide more in rodent models of myelin deficiencies (Kwiecien et al. [50]; Bu et al. [51]), one possibility is that decreased myelin levels in the *mdx* cerebral cortex could serve as a trigger for increased proliferation in oligodendrocyte progenitors. Alternatively, an increase in the “pipeline” of OPCs arriving in the corpus callosum due to increased oligodendrogenesis caused by neural stem cell dysfunction could contribute to increased numbers of proliferating OPCs in the developing white matter. The ability of dystrophin loss to alter proliferation is reminiscent of studies in which the loss of either dystrophin or dystroglycan resulted in abnormal proliferation phenotypes. For example, by using conditional knockout mice that lack dystroglycan in neural stem cells, our group found that dystroglycan regulates how neural stem cells proliferate and transition into gliogenic progenitors, such that dystroglycan loss resulted in *increased* stem cell proliferation and oligodendrogenesis, as well as increased proliferation in the OPCs themselves [26]. On the other hand, the loss of the Dp71 dystrophin isoform in PC12 neuronal cells has been shown to cause a *decrease* in proliferation by delaying G0/G1 transition. Here, Dp71 was postulated to be a component of the mitotic spindle and cytokinesis apparatus, as Dp71-depleted cells exhibited decreased lamin B1 and β-dystroglycan localization at the mitotic spindle and midbody in metaphase-anaphase or cytokinesis, as well as at the cleavage furrow in cytokinesis [52]. Finally, dystrophin loss in muscle stem cells, i.e., satellite cells, has been shown to cause a substantial decrease in asymmetric versus symmetric divisions by altering cell polarity and impairing mitotic spindle orientation [53]. Thus our new finding of increased numbers of proliferating cells in the developing white matter of *mdx* brains may reflect a failure of oligodendrocyte progenitors, less differentiated intermediate progenitors, or even the neural stem cells themselves, to divide appropriately.

Finally, while abnormal numbers of OPCs are present in *mdx* brains, the normal numbers of oligodendrocytes indicate that dystrophin loss does not prevent oligodendrocytes from reaching the white matter and surviving within it. Indeed, we did not find any significant change in oligodendroglial death in *mdx* corpus callosum. Dystrophin loss may instead hamper an oligodendrocyte’s ability to produce normal amounts of myelin or even to develop appropriate numbers of complex process branches, as was found in dystroglycan deficient oligodendrocytes [24]. Given that oligodendrocytes myelinate multiple axons, inefficient process extension and/or branching may slow myelination but not prevent it. Future studies will be needed to better understand dystrophin’s role in these and other cellular processes that contribute to successful myelination.

## Conclusions

Oligodendrocytes, the myelinating cells of the central nervous system, were found to express the Dp427, Dp140 and Dp71 dystrophin isoforms, as well as multiple dystrophin glyocoprotein complex members. We found that *mdx* mice, which lack Dp427 and serve as a mouse model for Duchenne muscular dystrophy (DMD), have delayed developmental myelination and in their cerebral cortices. We also found that cultured primary oligodendrocytes required dystrophin for appropriate and timely maturation, demonstrating that myelination delays in *mdx* brains were likely due, at least in part, to an oligodendrocyte-intrinsic requirement for dystrophin. Overall, the delayed myelination found in *mdx* mice is likely to have a negative impact on the speed and coordinated timing that is needed for complex brain processing, and may therefore contribute to DMD-related neurological deficits.

## Additional files

**Additional file 1: Figure S1.** Genotyping. Genotyping of tail DNA from male *mdx* and female *mdx*/+ control mice using a primer competition PCR as described in Shin et al. [30]. Arrows denote the typical products used to identify *mdx*/+ females and *mdx* males.

**Additional file 2: Figure S2.** CNP protein levels in the cerebral cortices of *mdx* and control mice. Quantification of mean CNP densitometries from western blots comparing protein lysates from cerebral cortices of control and *mdx* mice at p14, p21, 6wks and 2 months. Immunoblots to detect p115 were used as loading controls. CNP levels were not significantly different in *mdx* cerebral cortices with the exception of those at 2 months (**p < 0.01).

**Additional file 3: Figure S3.** MBP protein levels in the spinal cord and cerebellum of *mdx* and control mice. Quantification of mean MBP densitometries from western blots comparing protein lysates from spinal cord and cerebellum of control and *mdx* mice at p14, p21, 6wks and 2 months. Immunoblots to detect B-actin were used as loading controls. MBP levels were significantly different in *mdx* spinal cord at p21 and cerebellum at p14 (*p < 0.05).

**Additional file 4: Figure S4.** Oligodendrocytes in the corpus callosum of *mdx* and control mice have similar levels of cell death. (A) Quantification of mean TUNEL+ cells per mm^2^ in confocal images from p8 and p14 *mdx* and control corpus callosum. No significant changes were observed. (B) Quantification of mean CC1+ TUNEL+ cells per mm^2^ in confocal images from p8 and p14 *mdx* and control corpus callosum. No significant changes were observed. (C) Quantification of mean Enpp6+ TUNEL+ cells per mm^2^ in confocal images from p8 and p14 *mdx* and control corpus callosum. No significant changes were observed.

**Additional file 5: Figure S5.** Normal CNP levels in dystrophin-deficient oligodendrocytes. Western blot analysis of CNP protein in lysates obtained from differentiating oligodendrocytes at indicated days post-transfection with dystrophin (DMD) or control siRNA. Representative western blots are shown, including those for p115 as a loading control. CNP levels were similar in control and DMD siRNA transfected oligodendrocytes.

## Abbreviations

DGC: dystrophin glycoprotein complex
DMD: Duchenne muscular dystrophy
ECM: extracellular matrix
MBP: myelin basic protein
OPC: oligodendrocyte progenitor cell
PDL: poly-d-lysine

## References

[1] Wang Z, Chamberlain JS, Tapscott SJ, Storb R (2009). Gene therapy in large animal models of muscular dystrophy. ILAR J.

[2] Allikian MJ, McNally EM (2007). Processing and assembly of the dystrophin glycoprotein complex. Traffic.

[3] B Constantin (2014). Dystrophin complex functions as a scaffold for signalling proteins. Biochim Biophys Acta (BBA)-Biomembr.

[4] Pilgram GS, Potikanond S, Baines RA, Fradkin LG, Noordermeer JN (2010). The roles of the dystrophin-associated glycoprotein complex at the synapse. Mol Neurobiol.

[5] Haenggi T, Fritschy JM (2006). Role of dystrophin and utrophin for assembly and function of the dystrophin glycoprotein complex in non-muscle tissue. Cell Mol Life Sci.

[6] Blake DJ, Hawkes R, Benson MA, Beesley PW (1999). Different dystrophin-like complexes are expressed in neurons and glia. J Cell Biol.

[7] Blank M, Koulen P, Blake DJ, Kröger S (1999). Dystrophin and beta-dystroglycan in photoreceptor terminals from normal and mdx3Cv mouse retinae. Eur J Neurosci.

[8] Blitzblau R, Storer EK, Jacob MH (2008). Dystrophin and utrophin isoforms are expressed in glia, but not neurons, of the avian parasympathetic ciliary ganglion. Brain Res.

[9] Ricotti V, Mandy WP, Scoto M, Pane M, Deconinck N, Messina S, Mercuri E, Skuse DH, Muntoni F (2016). Neurodevelopmental, emotional, and behavioral problems in Duchenne muscular dystrophy in relation to underlying dystrophin gene mutations. Dev Med Child Neurol.

[10] Foran DR, Peterson AC (1992). Myelin acquisition in the central nervous system of the mouse revealed by an MBP-Lac Z transgene. J Neurosci.

[11] Baumann N, Pham-Dinh D (2001). Biology of oligodendrocyte and myelin in the mammalian central nervous system. Physiol Rev.

[12] Doorenweerd N, Straathof CS, Dumas EM, Spitali P, Ginjaar IB, Wokke BH, Schrans DG, Bergen JC, Zwet EW, Webb A, Buchem MA (2014). Reduced cerebral gray matter and altered white matter in boys with Duchenne muscular dystrophy. Ann Neurol.

[13] Moizard MP, Billard C, Toutain A, Berret F, Marmin N, Moraine C (1998). Are Dp71 and Dp140 brain dystrophin isoforms related to cognitive impairment in Duchenne muscular dystrophy?. Am J Med Genet Part A.

[14] Chamova T, Guergueltcheva V, Raycheva M, Todorov T, Genova J, Bichev S, Bojinova V, Mitev V, Tournev I, Todorova A (2013). Association between loss of Dp140 and cognitive impairment in Duchenne and Becker dystrophies. Balk J Med Genet.

[15] Felisari G, Boneschi FM, Bardoni A, Sironi M, Comi GP, Robotti M, Turconi AC, Lai M, Corrao G, Bresolin N (2000). Loss of Dp140 dystrophin isoform and intellectual impairment in Duchenne dystrophy. Neurology.

[16] Anderson JL, Head SI, Rae C, Morley JW (2002). Brain function in Duchenne muscular dystrophy. Brain.

[17] Miranda R, Laroche S, Vaillend C (2016). Reduced neuronal density in the CA1 anterodorsal hippocampus of the mdx mouse. Neuromuscul Disord.

[18] Goodnough CL, Gao Y, Li X, Qutaish MQ, Goodnough LH, Molter J, Wilson D, Flask CA, Yu X (2014). Lack of dystrophin results in abnormal cerebral diffusion and perfusion in vivo. Neuroimage.

[19] Xu S, Shi D, Pratt SJ, Zhu W, Marshall A (2015). Abnormalities in brain structure and biochemistry associated with mdx mice measured by in vivo MRI and high resolution localized 1 H MRS. Neuromuscul Disord.

[20] Relucio J, Tzvetanova ID, Ao W, Lindquist S, Colognato H (2009). Laminin alters fyn regulatory mechanisms and promotes oligodendrocyte development. J Neurosci.

[21] Chun SJ, Rasband MN, Sidman RL, Habib AA, Vartanian T (2003). Integrin-linked kinase is required for laminin-2-induced oligodendrocyte cell spreading and CNS myelination. J Cell Biol.

[22] Relucio J, Menezes MJ, Miyagoe-Suzuki Y, Takeda SI, Colognato H (2012). Laminin regulates postnatal oligodendrocyte production by promoting oligodendrocyte progenitor survival in the subventricular zone. Glia.

[23] Colognato H, Galvin J, Wang Z, Relucio J, Nguyen T, Harrison D, Yurchenco PD (2007). Identification of dystroglycan as a second laminin receptor in oligodendrocytes with a role in myelination. Development.

[24] Eyermann C, Czaplinski K, Colognato H (2012). Dystroglycan promotes filopodial formation and process branching in differentiating oligodendroglia. J Neurochem.

[25] Galvin J, Eyermann C, Colognato H (2010). Dystroglycan modulates the ability of insulin-like growth factor-1 to promote oligodendrocyte differentiation. J Neurosci Res.

[26] McClenahan FK, Sharma H, Shan X, Eyermann C, Colognato H (2016). Dystroglycan suppresses notch to regulate stem cell niche structure and function in the developing postnatal subventricular zone. Dev Cell.

[27] McCarthy KD, De Vellis J (1980). Preparation of separate astroglial and oligodendroglial cell cultures from rat cerebral tissue. J Cell Biol.

[28] Colognato H, Feltri ML (2005). Human diseases reveal novel roles for neural laminins. Trends Neurosci.

[29] Hu JG, Wu XJ, Feng YF, Xi GM, Deng LX, Wang ZH, Wang R, Shen L, Zhou JS, Lü HZ (2013). The molecular events involved in oligodendrocyte precursor cell proliferation induced by the conditioned medium from B104 neuroblastoma cells. Neurochem Res.

[30] Shin JH, Hakim CH, Zhang K, Duan D (2011). Genotyping mdx, mdx3cv and mdx4cv mice by primer competition PCR. Muscle Nerve.

[31] Bottenstein JE, Sato GH (1979). Growth of a rat neuroblastoma cell line in serum-free supplemented medium. Proc Natl Acad Sci.

[32] Colognato H, Baron W, Avellana-Adalid V, Relvas JB, Baron-Van Evercooren A, Georges-Labouesse E (2002). CNS integrins switch growth factor signalling to promote target-dependent survival. Nat Cell Biol.

[33] Imamura M, Ozawa E (1998). Differential expression of dystrophin isoforms and utrophin during dibutyryl-cAMP-induced morphological differentiation of rat brain astrocytes. Proc Natl Acad Sci.

[34] Villarreal-Silva M, Suárez-Sánchez R, Rodríguez-Muñoz R, Mornet D, Cisneros B (2010). Dystrophin Dp71 is critical for stability of the DAPs in the nucleus of PC12 cells. Neurochem Res.

[35] Fuentes-Mera L, Rodríguez-Muñoz R, González-Ramírez R, García-Sierra F, González E, Mornet D, Cisneros B (2006). Characterization of a novel Dp71 dystrophin-associated protein complex (DAPC) present in the nucleus of HeLa cells: members of the nuclear DAPC associate with the nuclear matrix. Exp Cell Res.

[36] González-Ramírez R, Morales-Lázaro SL, Tapia-Ramírez V, Mornet D, Cisneros B (2008). Nuclear and nuclear envelope localization of dystrophin Dp71 and dystrophin-associated proteins (DAPs) in the C2C12 muscle cells: DAPs nuclear localization is modulated during myogenesis. J Cell Biochem.

[37] Chaussenot R, Edeline JM, Le Bec B, El Massioui N, Laroche S, Vaillend C (2015). Cognitive dysfunction in the dystrophin-deficient mouse model of Duchenne muscular dystrophy: a reappraisal from sensory to executive processes. Neurobiol Learn Mem.

[38] Remmelink E, Aartsma-Rus A, Smit AB, Verhage M, Loos M, van Putten M (2016). Cognitive flexibility deficits in a mouse model for the absence of full-length dystrophin. Genes Brain Behav.

[39] Vaillend C, Billard JM, Claudepierre T, Rendon A, Dutar P, Ungerer A (1998). Spatial discrimination learning and CA1 hippocampal synaptic plasticity in mdx and mdx3cv mice lacking dystrophin gene products. Neuroscience.

[40] Romo-Yáñez J, Montañez C, Salazar-Olivo LA (2011). Dystrophins and DAPs are expressed in adipose tissue and are regulated by adipogenesis and extracellular matrix. Biochem Biophys Res Commun.

[41] Nichols B, Takeda SI, Yokota T (2015). Nonmechanical roles of dystrophin and associated proteins in exercise, neuromuscular junctions, and brains. Brain Sci.

[42] Keefe AC, Kardon G (2015). A new role for dystrophin in muscle stem cells. Nat Med.

[43] Batchelor CL, Winder SJ (2006). Sparks, signals and shock absorbers: how dystrophin loss causes muscular dystrophy. Trends Cell Biol.

[44] Tadayoni R, Rendon A, Soria-Jasso LE (2012). Dystrophin Dp71: the smallest but multifunctional product of the Duchenne muscular dystrophy gene. Mol Neurobiol.

[45] Miller G, Moore CJ, Terry R, La Riviere T, Mitchell A, Piggott R, Dear TN, Wells DJ, Winder SJ (2012). Preventing phosphorylation of dystroglycan ameliorates the dystrophic phenotype in mdx mouse. Hum Mol Genet.

[46] Sotgia F, Lee H, Bedford MT, Petrucci T, Sudol M, Lisanti MP (2001). Tyrosine phosphorylation of β-dystroglycan at its WW domain binding motif, PPxY, recruits SH2 domain containing proteins. Biochemistry.

[47] Domingues HS, Portugal CC, Socodato R, Relvas JB (2016). Oligodendrocyte, astrocyte, and microglia crosstalk in myelin development, damage, and repair. Front Cell Dev Biol..

[48] Früh S, Romanos J, Panzanelli P, Bürgisser D, Tyagarajan SK, Campbell KP, Santello M, Fritschy JM (2016). Neuronal dystroglycan is necessary for formation and maintenance of functional CCK-positive basket cell terminals on pyramidal cells. Journal of Neuroscience..

[49] Morita J, Kano K, Kato K, Takita H, Sakagami H, Yamamoto Y, Mihara E, Ueda H, Sato T, Tokuyama H, Arai H (2016). Structure and biological function of ENPP6, a choline-specific glycerophosphodiester phosphodiesterase. Sci Rep..

[50] Kwiecien JM (2010). Cellular compensatory mechanisms in the CNS of dysmyelinated rats. Comp Med..

[51] Bu J, Banki A, Wu Q, Nishiyama A (2004). Increased NG2+ glial cell proliferation and oligodendrocyte generation in the hypomyelinating mutant shiverer. Glia..

[52] Villarreal-Silva M, Centeno-Cruz F, Suárez-Sánchez R, Garrido E, Cisneros B (2011). Knockdown of dystrophin Dp71 impairs PC12 cells cycle: localization in the spindle and cytokinesis structures implies a role for Dp71 in cell division. PLoS ONE.

[53] Dumont NA, Wang YX, Von Maltzahn J, Pasut A, Bentzinger CF, Brun CE, Rudnicki MA (2015). Dystrophin expression in muscle stem cells regulates their polarity and asymmetric division. Nat Med.

