# Supplementary figures and images for: Myelination is delayed during postnatal brain development in the mdx mouse model of Duchenne muscular dystrophy
Source: BMC Neurosci. 2017 Aug 14;18:63. doi: 10.1186/s12868-017-0381-0 (PMC5556620; doi:10.1186/s12868-017-0381-0)

**Figure S1**

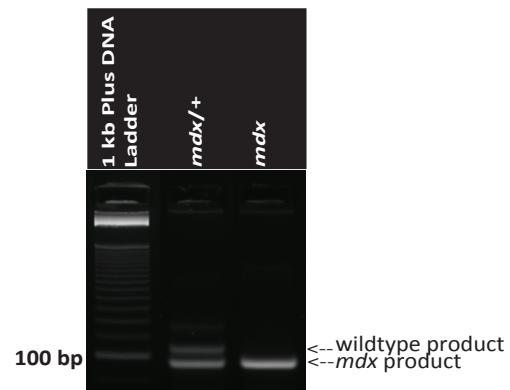

Supplement: Supplementary file 1 — Additional file 1: Figure S1. Genotyping. Genotyping of tail DNA from male mdx and female mdx/+ control mice using a primer competition PCR as described in Shin et al. [30]. Arrows denote the typical products used to identify mdx/+ females and mdx males. [file 12868_2017_381_MOESM1_ESM.pdf]

Figure S2

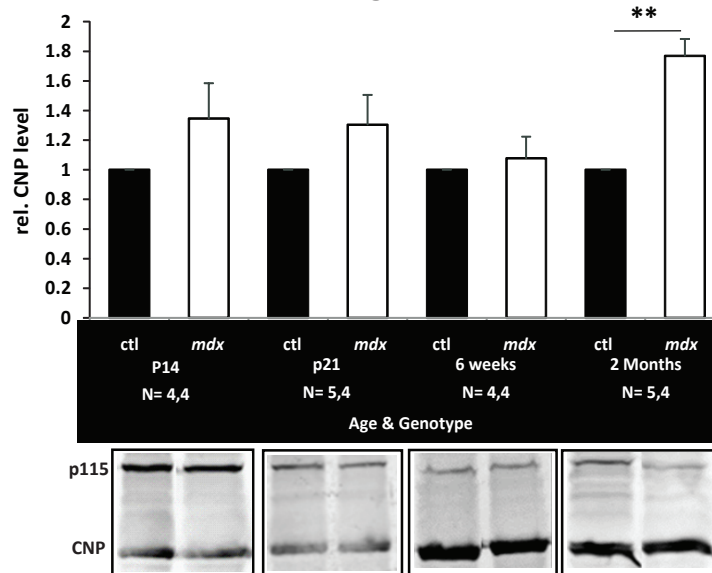

Supplement: Supplementary file 2 — Additional file 2: Figure S2. CNP protein levels in the cerebral cortices of mdx and control mice. Quantification of mean CNP densitometries from western blots comparing protein lysates from cerebral cortices of control and mdx mice at p14, p21, 6wks and 2 months. Immunoblots to detect p115 were used as loading controls. CNP levels were not significantly different in mdx cerebral cortices with the exception of those at 2 months (**p < 0.01). [file 12868_2017_381_MOESM2_ESM.pdf]

Figure S3

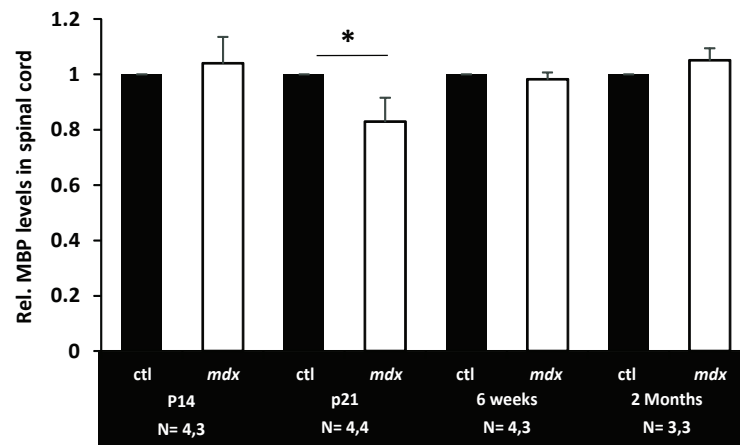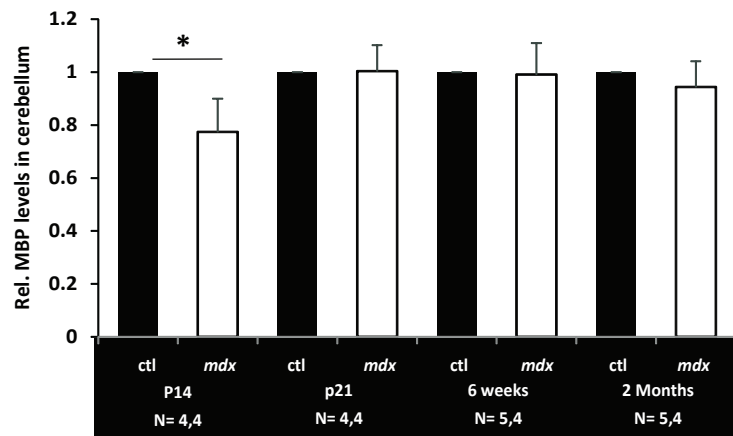

Supplement: Supplementary file 3 — Additional file 3: Figure S3. MBP protein levels in the spinal cord and cerebellum of mdx and control mice. Quantification of mean MBP densitometries from western blots comparing protein lysates from spinal cord and cerebellum of control and mdx mice at p14, p21, 6wks and 2 months. Immunoblots to detect B-actin were used as loading controls. MBP levels were significantly different in mdx spinal cord at p21 and cerebellum at p14 (*p < 0.05). [file 12868_2017_381_MOESM3_ESM.pdf]

Figure S4

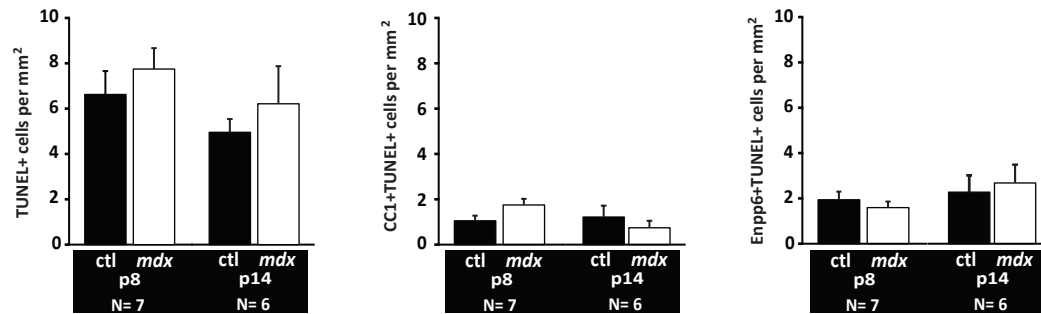

Supplement: Supplementary file 4 — Additional file 4: Figure S4. Oligodendrocytes in the corpus callosum of mdx and control mice have similar levels of cell death. (A) Quantification of mean TUNEL+ cells per mm2 in confocal images from p8 and p14 mdx and control corpus callosum. No significant changes were observed. (B) Quantification of mean CC1+ TUNEL+ cells per mm2 in confocal images from p8 and p14 mdx and control corpus callosum. No significant changes were observed. (C) Quantification of mean Enpp6+ TUNEL+ cells per mm2 in confocal images from p8 and p14 mdx and control corpus callosum. No significant changes were observed. [file 12868_2017_381_MOESM4_ESM.pdf]

Figure S5

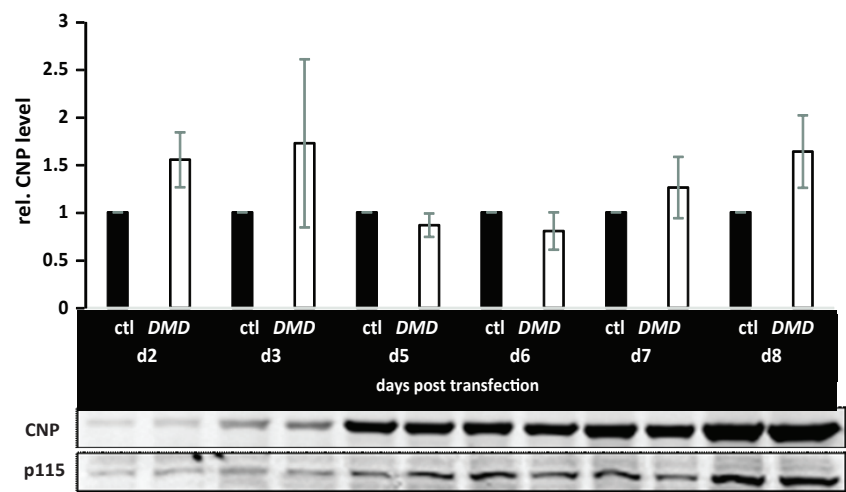

Supplement: Supplementary file 5 — Additional file 5: Figure S5. Normal CNP levels in dystrophin-deficient oligodendrocytes. Western blot analysis of CNP protein in lysates obtained from differentiating oligodendrocytes at indicated days post-transfection with dystrophin (DMD) or control siRNA. Representative western blots are shown, including those for p115 as a loading control. CNP levels were similar in control and DMD siRNA transfected oligodendrocytes. [file 12868_2017_381_MOESM5_ESM.pdf]
